# Serum Calcification Propensity T50 Associates with Disease Severity in Patients with Pseudoxanthoma Elasticum

**DOI:** 10.3390/jcm11133727

**Published:** 2022-06-28

**Authors:** Lukas Nollet, Matthias Van Gils, Suzanne Fischer, Laurence Campens, Swapna Karthik, Andreas Pasch, Julie De Zaeytijd, Bart P. Leroy, Daniel Devos, Tine De Backer, Paul J. Coucke, Olivier M. Vanakker

**Affiliations:** 1 Center for Medical Genetics, Ghent University Hospital, 9000 Ghent, Belgium; lukas.nollet@ugent.be (L.N.); matthias.vangils@ugent.be (M.V.G.); bart.leroy@ugent.be (B.P.L.); paul.coucke@ugent.be (P.J.C.); 2 Department of Biomolecular Medicine, Ghent University, 9000 Ghent, Belgium; 3 Ectopic Mineralization Research Group Ghent, 9000 Ghent, Belgium; 4 Laboratory of Experimental Cancer Research, Ghent University, 9000 Ghent, Belgium; suzanne.fischer@ugent.be; 5 Department of Cardiology, Ghent University Hospital, 9000 Ghent, Belgium; laurence.campens@ugent.be (L.C.); tine.debacker@ugent.be (T.D.B.); 6 Calciscon AG, 2560 Nidau, Switzerland; swapna.karthik@gmx.ch (S.K.); andreas.pasch@calciscon.com (A.P.); 7 Institute of Physiology and Pathophysiology, Johannes Kepler University Linz, 4040 Linz, Austria; 8 Department of Nephrology, Lindenhofspital, 3012 Bern, Switzerland; 9 Practice for Internal Medicine and Nephrology at Hirschengraben, 3011 Bern, Switzerland; 10 Department of Ophthalmology, Ghent University Hospital, 9000 Ghent, Belgium; julie.dezaeytijd@ugent.be; 11 Division of Ophthalmology, The Children’s Hospital of Philadelphia, Philadelphia, PA 19104, USA; 12 Department of Radiology, Ghent University Hospital, 9000 Ghent, Belgium; daniel.devos@uzgent.be

**Keywords:** pseudoxanthoma elasticum, PXE, ectopic calcification, biomarker, genetics, rare diseases, serum calcification propensity T50

## Abstract

Pseudoxanthoma elasticum (PXE) is a currently intractable genetic disorder characterized by progressive ectopic calcification in the skin, eyes and arteries. Therapeutic trials in PXE are severely hampered by the lack of reliable biomarkers. Serum calcification propensity T50 is a blood test measuring the functional anticalcifying buffer capacity of serum. Here, we evaluated T50 in PXE patients aiming to investigate its determinants and suitability as a potential biomarker for disease severity. Fifty-seven PXE patients were included in this cross-sectional study, and demographic, clinical, imaging and biochemical data were collected from medical health records. PXE severity was assessed using Phenodex scores. T50 was measured using a validated, nephelometry-based assay. Multivariate models were then created to investigate T50 determinants and associations with disease severity. In short, the mean age of patients was 45.2 years, 68.4% was female and mean serum T50 was 347 min. Multivariate regression analysis identified serum fetuin-A (*p* < 0.001), phosphorus (*p* = 0.007) and magnesium levels (*p* = 0.034) as significant determinants of T50, while no correlations were identified with serum calcium, eGFR, plasma PPi levels or the *ABCC6* genotype. After correction for covariates, T50 was found to be an independent determinant of ocular (*p* = 0.013), vascular (*p* = 0.013) and overall disease severity (*p* = 0.016) in PXE. To conclude, shorter serum T50—indicative of a higher calcification propensity—was associated with a more severe phenotype in PXE patients. This study indicates, for the first time, that serum T50 might be a clinically relevant biomarker in PXE and may thus be of importance to future therapeutic trials.

## 1. Introduction

Systemic ectopic calcification is a pathologic process resulting from a chronic imbalance between pro- and antimineralizing factors, giving rise to an uncontrolled deposition of calcium phosphate crystals in soft tissues [1]. Previously thought to be a passive process without any relevant clinical effects, recent insights have proven the contrary, with an abundance of cellular targets and pathways involved in this highly regulated and active mechanism that leads to micro- and macroscopic calcification with consequent clinical symptoms and organ dysfunction [2]. Ectopic calcification, in particular medial vascular calcification, is the main underlying pathophysiological process in some of the most frequent Western disorders, such as arteriosclerosis, imposing a high burden at a population level [3,4]. Furthermore, systemic ectopic calcification causes increased arterial stiffness, heart failure and chronic limb-threatening ischemia, and is a well-established independent risk factor for premature death in patients with diabetes mellitus or chronic kidney disease (CKD) [5,6].

An adequate assessment of systemic ectopic calcification propensity in individual patients may significantly enhance risk stratification, personalized treatment and patient prognosis [7]. The T50 test determines the intrinsic calcification propensity of human serum by measuring the delay of the transformation from primary calciprotein particles (CPPs)—circulating clusters of amorphous calcium phosphate and mineral-buffering proteins such as fetuin-A or Gla-rich protein—to secondary, crystalline CPPs [8,9,10]. A high T50 (half-maximal transformation time point) value indicates that the serum sample is highly resistant to calcification, whereas a low T50 value indicates calcification-prone serum [10]. Since its introduction by Pasch et al. in 2012, the T50 test has been implemented in a variety of patient cohorts in clinical research settings, including type 1 and 2 diabetes mellitus, CKD and hemodialysis patients, as well as in healthy individuals [11,12,13,14]. T50 has been shown to be an independent predictor of cardiovascular events and all-cause mortality in CKD and renal transplantation patients, associated with glycemic control in type 2 diabetes patients and adequately predicted the risk of cardiovascular death in a large general population-based cohort [12,14,15,16]. Interestingly, therapeutic interventions, including oral magnesium supplementation in CKD patients or the use of citric-acid-buffered dialysis solution in individuals receiving hemodialysis have proven effective in ameliorating T50 values [17,18]. However, whether treatment-induced changes in T50 also result in more favorable clinical outcomes needs yet to be determined.

Pseudoxanthoma elasticum (PXE, OMIM #264800) is considered a paradigm for hereditary ectopic calcification disorders and is caused by biallelic pathogenic variants in the *ABCC6* gene [19]. *ABCC6* encodes a transmembrane ATP-dependent efflux transporter primarily expressed in the liver and kidneys, transferring a still elusive substrate [20,21]. PXE patients develop mineralization of elastic fibers in the mid-dermis of the skin (with papular skin lesions and excessive skin folds), Bruch membrane of the eye (predisposing to choroidal neovascularization, hemorrhage and vision loss) and arterial media [22]. The latter results in cardiovascular disease with a higher risk for coronary (myocardial infarction), cerebral (stroke) and peripheral artery disease [23,24]. To date, no effective treatment options are available for PXE patients. As the chronic and slowly progressive nature of the disease impedes the use of conventional clinical or radiological endpoints in therapeutic trials, translational research into novel disease-modifying drugs is currently severely hampered by the lack of specific biomarkers.

In this study, we, therefore, evaluated serum calcification propensity T50 in PXE patients, aiming to investigate its determinants and suitability as a potential biomarker for disease severity in this at-risk population.

## 2. Materials and Methods

### 2.1. Study Population and Patient Data Collection

Participants were recruited from the PXE Clinic of the Center for Medical Genetics of Ghent University Hospital, Belgium. All participants (n = 57) had a clinically and genetically confirmed diagnosis of PXE. The study was approved by the Ethics Committee of the Ghent University Hospital and the Declaration of Helsinki protocols were followed. Written informed consent was obtained from all participants. Data collection was performed using the participants’ medical health records. Both demographic, clinical, imaging and biochemical data were collected. Ophthalmological examination, including funduscopy, was performed by medical retina specialists of the department of Ophthalmology at Ghent University Hospital. Regarding cardiovascular risk factors, arterial hypertension was defined as systolic blood pressure > 140 mmHg and diastolic blood pressure > 90 mmHg during repeated measurements or use of antihypertensive medication, and hypercholesterolemia was defined as fasting LDL-C > 115 mg/dL, total cholesterol > 200 mg/dL or the use of lipid-lowering drugs [25]. Diabetes mellitus was defined as fasting glucose > 126 mg/dL or the use of antidiabetic medication [25]. Laboratory measurements of serum minerals (calcium, magnesium and phosphorus), alkaline phosphatase and kidney function (creatinine, eGFR calculated according to the CKD-EPI formula) were performed at the department of Laboratory Medicine of the Ghent University Hospital according to standard protocols. Inorganic pyrophosphate (PPi) was measured in platelet-free plasma obtained from citrate blood tubes, using an ATP sulfurylase-based assay with bioluminescent detection as described by Sanchez-Tevar et al. [26]. Fetuin-A was measured in serum samples using a commercially available ELISA assay (Proteintech, IL, USA) according to manufacturer’s instructions.

Sequencing and multiplex ligation-dependent probe amplification (MLPA) of the *ABCC6* gene was performed as previously described [27]. Variants were then classified according to Verschuere et al. [28]. Genotype groups were defined as follows: (I) biallelic loss-of-function variants (i.e., nonsense and/or frameshift variants and/or (multi-)exon deletions), (II) compound heterozygous loss-of-function + missense variant, (III) biallelic missense variants and (IV) combinations including splice site variants or single *ABCC6* variant. The latter category was excluded from correlation analysis between *ABCC6* genotype and T50 value, as it could not be determined whether these splice-site variants led to a less functional or to an absent protein.

### 2.2. Assessment of PXE Disease Severity

PXE disease severity was assessed using the Phenodex-FlorMore scoring system as described by Boraldi et al., evaluating the extent of skin (S0–S3), eye (E0–E4), gastrointestinal (G0–G1), vascular (V0–V3), cardiac (C0–C2) and renal (R0–R1) manifestations [29]. Higher Phenodex scores reflected more severe clinical manifestations.

### 2.3. Measurement of cIMT, cfPWV and Total Body CT Calcium Score

Vascular measurements were performed using a commercially available ultrasound system (Vivid 7, GE Medical Systems, Oslo, Norway) equipped with a vascular transducer set at 10 MHz. After 10 min of rest, two-dimensional and tissue Doppler images of the common carotid and femoral arteries were obtained. Carotid intima–media thickness (cIMT) and carotid–femoral pulse wave velocity (cfPWV) were then calculated as previously described [23]. Noncontrast-enhanced whole-body CT scanning was performed in 14 PXE patients using a Siemens Somatom Definition Flash dual source CT scanner (Erlangen, Germany). Calcium scoring in large- and medium-sized arterial vessels (i.e., coronary arteries, thoracic and abdominal aorta, left and right iliac, femoral, popliteal and crural arteries) was performed on a Siemens workstation with a threshold of 130 Hounsfield units (HU); only lesions with an area ≥ 3 pixels were withheld by the software. Agatston calcium scores were determined as previously described [30]. A cumulative total body CT calcium score was then calculated by adding up all Agatston scores of the aforementioned vascular regions.

### 2.4. Measurement of Serum Calcification Propensity T50

Serum samples for T50 measurement were obtained from the participants through venipuncture at the same moment when blood sampling for routine blood chemistry and PPi was performed. Serum samples were stored at −80 °C without thawing. T50 calcification propensity measurements were performed at Calciscon AG, Nidau, Switzerland, as previously described [10]. In short, serum samples were thawed, vortexed and centrifuged after which they were supersaturated with calcium and phosphate solutions (pH 7.40). Pipetting was performed using an automated high-precision pipetting system (Freedom EVO 100, Tecan, Switzerland). Samples were then measured in triplicate in 96-well plates at 37 °C for 600 min in a Nephelostar nephelometer (BMG Labtech, Ortenberg, Germany), hence, live-monitoring the transformation of primary to secondary CPPs in the different samples (batch 1: n = 23; batch 2: n = 34). To determine the half-maximal transformation time (T50), data analyses of nonlinear regression curves were performed using the Calciscon T50 Analysis Software. Intra- and interassay coefficients of variation were 2.2% and 3.4%, respectively.

### 2.5. Statistical Analysis

Continuous data are presented as mean ± standard deviation (SD) if normally distributed or as median and interquartile range (IQR) otherwise. Categorical data are presented as absolute count and corresponding percentages. Normality testing was performed using the Shapiro–Wilk test. For descriptive and comparative purposes, patient baseline characteristics were stratified by T50 tertile. Means or medians were compared between T50 tertile groups using analysis of variance (ANOVA) testing with Tukey’s post hoc correction for multiple comparisons, or Kruskal–Wallis test, as appropriate. Categorical variables were compared using chi-squared test. Pearson or Spearman correlation coefficients were used to investigate correlations between baseline variables and T50, as appropriate. Main determinants of T50 were evaluated using a stepwise multivariate linear regression model in which only variables that were significantly associated with T50 upon initial univariate analysis with T50 as dependent variable were included. Likewise, associations between T50 and clinical outcomes (i.e., Phenodex disease severity scores or radiological markers, including cIMT, cfPWV and CT calcium score) were investigated using multivariate ordinal (Phenodex scores) or linear (cIMT, cfPWV or CT calcium score) regression analysis with T50 and other predicting baseline variables (as identified using univariate analysis) as covariates. Regression models were checked for absence of multicollinearity. A *p*-value < 0.05 was considered statistically significant. All statistical analyses were performed using IBM SPSS Statistics (version 27) and graphs were created using GraphPad Prism (version 9.2).

## 3. Results

### 3.1. Baseline Characteristics and Serum T50 in PXE Patients

Fifty-seven PXE patients were included in the current study (Supplemental Table S1). Patient baseline characteristics, stratified by T50 tertile, are shown in Table 1.

In short, the mean age was 45.2 ± 15.7 years and 68.4% was female. Regarding biochemical parameters, all PXE patients had severely reduced plasma PPi levels (mean: 0.50 ± 0.16 µmol/L). Normal kidney function (eGFR > 90 mL/min; CKD stage 1) or mild renal impairment (eGFR 60–89 mL/min, CKD stage 2) was present in 56.1% and 40.4% of patients, respectively, while moderate CKD stage 3A (eGFR 45–59 mL/min) was found in 3.5% of cases. None of the patients had moderate, severe or end-stage CKD 3B-5 (eGFR < 44 mL/min).

**Figure 1:**
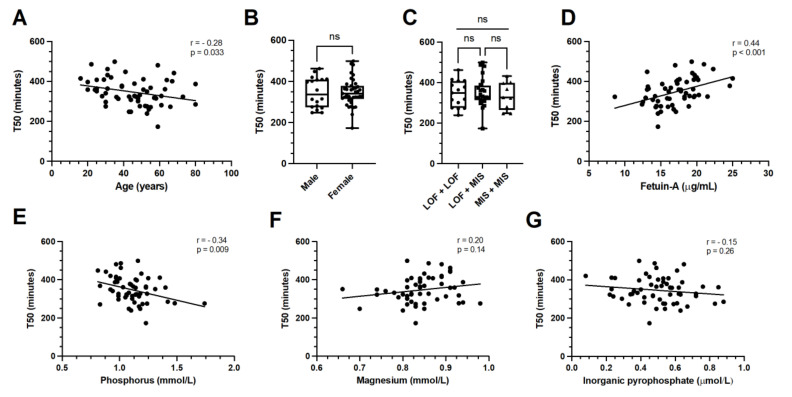
Associations of baseline characteristics with serum calcification propensity T50 in PXE. T50 significantly correlated with age (panel (**A**)), but not with sex or *ABCC6* genotype (panels (**B**,**C**)). Serum fetuin-A levels correlated strongly and positively with T50 (panel (**D**)). An inverse correlation existed between T50 and serum phosphorus levels (panel (**E**)), while only a trend towards correlation was identified with serum magnesium levels in univariate analysis (panel (**F**)). Plasma inorganic pyrophosphate did not significantly correlate with serum T50 in PXE patients (panel (**G**)). LOF—loss-of-function variants; MIS—missense variants; ns—non significant.

Serum T50 values were normally distributed throughout the patient cohort (Shapiro–Wilk: *p* = 0.79) with a mean value of 347 ± 68 min (range: 173–499 min); the middle T50 tertile ranged from 316 to 368 min. While the mean age was not found to be significantly different across tertiles (*p* = 0.17), Pearson’s correlation coefficient did show a significant inverse correlation between age and T50 (r = −0.28; *p* = 0.033), indicating a progressive decrease in the T50 value with advancing age (Figure 1A).

A female predominance was present in the second T50 tertile compared with the first and third tertile (*p* = 0.010), but no significant overall correlation was identified (r = −0.01; *p* = 0.94) (Figure 1B). Traditional cardiovascular risk factors, including current smoking, arterial hypertension, hypercholesterolemia, diabetes mellitus and BMI, were not significantly associated with T50 (*p* > 0.05). Regarding blood chemistry parameters, a significant inverse correlation was identified between serum phosphorus levels and T50, with high phosphorus concentrations associating with low T50 values (r = −0.34; *p* = 0.009) (Figure 1E). Additionally, serum fetuin-A was found to be significantly and strongly correlated with T50 values in PXE patients (r = 0.44; *p* < 0.001) (Figure 1D). No significant differences between T50 tertiles were observed for serum calcium, magnesium, alkaline phosphatase or plasma PPi levels (*p* > 0.05) (Figure 1F,G). Similarly, kidney function parameters, including creatinine and eGFR, did not significantly correlate with T50.

Regarding the *ABCC6* genotype, combinations of loss-of-function and missense variants were observed in 42.1% of cases, while biallelic loss-of-function variants were present in 26.3% of patients (Table 1). Biallelic missense variants and combinations including splice-site variants or single *ABCC6* variant, were identified in both 15.8% of cases. Overall, no significant difference was observed between *ABCC6* genotypes across T50 tertiles (*p* > 0.05) (Figure 1C).

**Figure 2:**
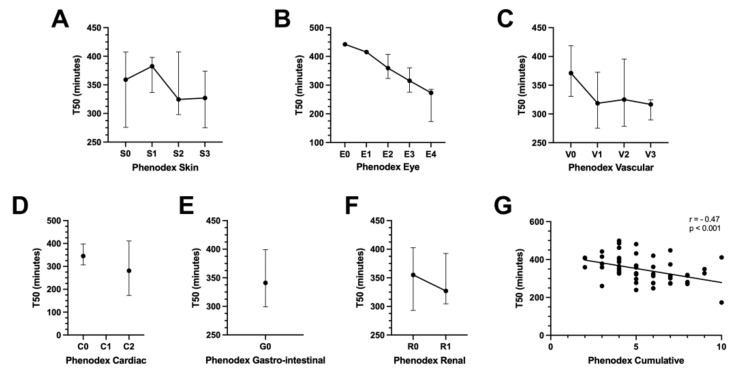
Associations of Phenodex disease severity scores with serum calcification propensity T50 in PXE. Serum T50 inversely correlated with Phenodex eye (panel (**B**)), vascular (panel (**C**)) and cumulative (panel (**G**)) scores in PXE patients. No correlations were found with Phenodex skin (panel (**A**)), cardiac, gastrointestinal or renal scores (panels (**D**–**F**)). Graphs represent median values and interquartile range (error bar).Data on cIMT (n = 16), cfPWV (n = 14) and total body CT calcium score (n = 14) were available in a subset of PXE patients (median age: 45.0 years (IQR: 31.0–58.5 years), 68.8% female), showing a significant inverse correlation between cIMT and T50 (r = −0.57; *p* = 0.022) and cfPWV and T50 (r = −0.61; *p* = 0.020), while no significant association with T50 was found for the total body CT calcium score (r = −0.45; *p* = 0.10) (Supplemental Figure S1).

To evaluate the extent of disease severity in PXE patients, the Phenodex-FlorMore scoring system was used, revealing a median S (skin) score of 2 (IQR: 1.5–2), E (eye) score of 2 (2–3), V (vascular) score of 1 (0–1), C (cardiac) score of 0 (0–0) and R (renal) score of 0 (0–1). All patients had a G (gastrointestinal) score of 0. The median cumulative Phenodex score was five (4–7). Significant and strong associations with T50 were observed for the Phenodex E score (r = −0.47; *p* < 0.001), Phenodex V score (r = −0.38; *p* = 0.003) and Phenodex cumulative score (r = −0.47; *p* < 0.001) (Figure 2B,C,G).

### 3.2. Determinants of Serum T50 in PXE Patients

Determinants of calcification propensity T50 were evaluated using multivariate linear regression analysis identifying serum fetuin-A (standardized β = 0.40; *p* < 0.001), serum phosphorus (standardized β = −0.32; *p* = 0.007) and serum magnesium levels (standardized β = 0.25; *p* = 0.034) as independent determinants of T50 in PXE patients (adjusted R^2^ = 0.30; ANOVA: *p* < 0.001) (Table 2).

### 3.3. Associations of Serum T50 with PXE Disease Severity

To assess whether PXE disease severity could be predicted by serum T50 after correction for covariates, a stepwise multivariate ordinal regression model was created using the Phenodex score as a dependent variable. The final models are detailed in Table 3.

Regarding the Phenodex E score, significant correlations were found with age (*p* < 0.001), T50 (*p* < 0.001), serum fetuin-A (*p* < 0.001), serum phosphorus (*p* = 0.030), eGFR (*p* = 0.010) and plasma PPi (*p* = 0.036) in the univariate analysis, while only age (estimate = 0.084; *p* = 0.001), T50 (estimate = −0.014; *p* = 0.013) and serum fetuin-A (estimate = −0.25; *p* = 0.044) remained significant in the multivariate model. Similar results were obtained for the Phenodex V score, for which significant univariate correlations were identified with age (*p* = 0.008), serum fetuin-A (*p* = 0.009), T50 (*p* = 0.013), eGFR (*p* = 0.029) and the presence of arterial hypertension (*p* = 0.045). In the multivariate model, only T50 (estimate = −0.010; *p* = 0.013) and arterial hypertension (estimate = 1.42; *p* = 0.039) remained significantly associated with the Phenodex V score after correction for covariates. Regarding the Phenodex cumulative score, significant univariate correlations were found with age (*p* = 0.017), serum fetuin-A (*p* = 0.011) and T50 (*p* < 0.001), while only T50 (estimate = −0.010; *p* = 0.016) remained significant in the multivariate model. Finally, serum T50 was not found to be an independent determinant of the Phenodex skin, cardiac or renal scores after multivariate regression analysis (*p* > 0.05). As the Phenodex gastrointestinal score only had values of “0” in the studied patient cohort, no further analysis was performed.

### 3.4. Associations of Serum T50 with Vascular Ultrasound Measurements and CT Calcium Score in PXE

Concerning cIMT in PXE patients, univariate analysis identified significant correlations with age (*p* = 0.013), T50 (*p* = 0.022) and the presence of arterial hypertension (*p =* 0.020), while only T50 (standardized β = −0.50; *p* = 0.016) and arterial hypertension (standardized β = 0.51; *p* = 0.015) remained significant in the multivariate model (Supplemental Table S2). Similar results were obtained for cfPWV, for which significant univariate correlations were identified with age (*p* < 0.001), sex (*p* = 0.034), T50 (*p* = 0.020) and eGFR (*p <* 0.001). After correction for confounders, only T50 (standardized β = −0.32; *p* = 0.042) and eGFR (standardized β = −0.67; *p* < 0.001) remained significantly associated with cfPWV (Supplemental Table S2). Finally, multivariate regression analysis did not identify T50 as a significant determinant of the total body CT calcium score in PXE patients (*p* > 0.05).

## 4. Discussion

Since the discovery of its causal gene *ABCC6* more than 20 years ago, significant advances have been attained in unravelling the molecular and clinical pathogenesis of PXE, a multisystemic Mendelian ectopic calcification disorder causing severe visual impairment, skin lesions and vascular complications. While promising novel treatment options, such as exogenous PPi, bisphosphonates, alkaline phosphatase inhibitors or minocycline, have all proven effective in in vitro or laboratory animal model systems, translation towards clinical trials in human PXE patients remains impaired by the lack of reliable biomarkers for disease severity, particularly as PXE disease progression is relatively slow [31,32,33].

**Figure 3:**
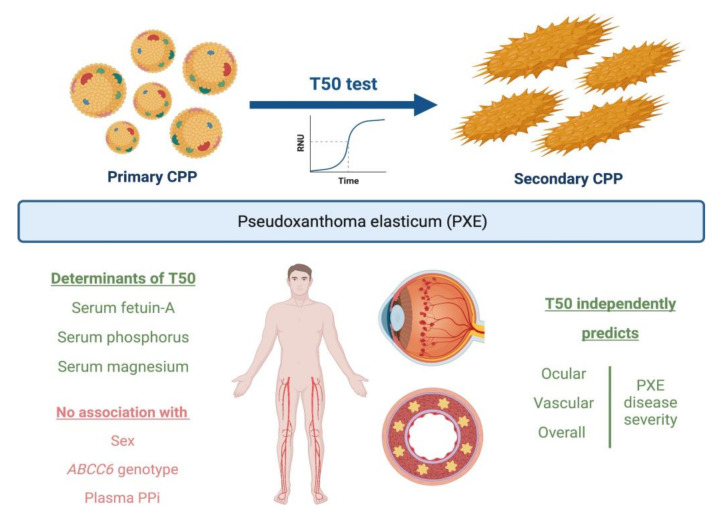
Graphical overview of study results on the role of serum calcification propensity T50 in PXE. CPP—calciprotein particle; PPi—inorganic pyrophosphate.

In this cross-sectional study, we therefore investigated the association between serum calcification propensity T50 and PXE disease severity as evaluated using the Phenodex scoring system in a group of 57 PXE patients followed at the Ghent Center for Medical Genetics. Briefly, we found that shorter serum T50—indicative of a higher calcification propensity—was an independent determinant of increased ocular, vascular and overall disease severity in PXE patients (Figure 3).

Similar to the results from previous studies investigating associations with T50 in other patient populations, we found that serum fetuin-A and serum phosphorus levels were important and independent determinants of T50 in individuals with PXE, with higher fetuin-A and lower phosphorus concentrations resulting in higher, that is, better, T50 values [14,34]. Fetuin-A, a phosphorylated liver glycoprotein and potent systemic calcification inhibitor, is known to be significantly reduced in the serum of *Abcc6−/−* mice and human PXE patients, while restoring normal serum fetuin-A levels rescued the ectopic calcification phenotype in *Abcc6−/−* mice [35,36,37]. However, the precise pathomechanism linking *ABCC6* deficiency to reduced fetuin-A levels has not yet been elucidated.

Interestingly, serum calcium levels were not found to be associated with T50 in PXE patients—contrary to earlier findings in patients with CKD [34]—which may potentially be attributed to the fact that all PXE patients in the current study had serum calcium levels within the normal reference range and that dysfunctional calcium homeostasis is not known to be involved in the pathogenesis of PXE, as opposed to CKD.

In this study, the serum magnesium level was also identified through multivariate modeling as a significant determinant of T50 in PXE patients, which is in accordance with earlier findings in both healthy and diabetic individuals [11,14]. The role of magnesium as a potential anticalcifying agent in PXE has been controversial with preclinical studies in *Abcc6−/−* mice showing that an increased magnesium intake substantially reduced ectopic calcification and cIMT in these animals, while a randomized clinical trial involving 44 PXE patients failed to identify a significant improvement of the skin phenotype following a 2-year period of oral magnesium supplementation [38,39,40]. However, as the reliability and validity of primary endpoints used in this trial were questionable, evaluating prospective changes in T50 in both placebo- and magnesium-treated PXE patients might be of interest.

No significant correlations between T50 and plasma PPi levels were identified in our current study. Circulating PPi—originating from ABCC6-mediated ATP release and the subsequent breakdown by ENPP1—is known to be significantly reduced in PXE patients compared with healthy controls, and is a potent endogenous inhibitor of calcium crystal formation [41]. We may hypothesize that the absent correlation between PPi and T50 in this study might be attributed to (I) high intra- and interindividual variability in circulating plasma PPi (e.g., fasting versus nonfasting, diurnal changes, females versus males), (II) the effect of PPi levels on T50 was outweighed by the much larger effect of inorganic phosphate (Pi), as the intrinsic calcification propensity is mostly dependent on the Pi/PPi ratio, or (III) technical variance in the bioluminescent assay used to determine PPi concentration, which is notoriously difficult to measure in biological samples. Larger cohort studies measuring PPi prospectively and repeatedly are, therefore, needed to further investigate a potential link between PPi and T50 in PXE patients.

In our current study, serum calcification propensity T50 was found to be independently and inversely associated with Phenodex eye, vascular and cumulative scores, and might, therefore, be a promising and clinically relevant biomarker for PXE disease severity. In their large general population-based study, Eelderinck et al. already hypothesized that the predictive value of T50 might be higher in specific patient subgroups who are at increased risk of developing systemic ectopic calcification, as they demonstrated more pronounced associations between T50 and cardiovascular mortality in individuals with type 2 diabetes mellitus compared to nondiabetic individuals [14]. As PXE patients have an intrinsically high risk of developing calcification-related complications due to their *ABCC6*-deficient genotype, the prognostic value of serum T50 might even be higher in this distinct patient population.

As for the Phenodex skin scores, the absence of significant associations with T50 in the current study might be attributed to the fact that the severity of cutaneous alterations in PXE is only partially reflected by the Phenodex S score as, for example, redundant skin folds due to loss of elasticity might not always reflect higher disease activity compared to extensive papules or plaques. Future studies should, therefore, focus on novel evaluation and grading methods of skin lesion severity, for example, by implementing in vivo autofluorescence and dermoscopy techniques [42,43]. Potential associations between Phenodex cardiac and gastrointestinal scores and T50 could not be adequately evaluated in our current PXE patient population due to the low prevalence of Phenodex-scored events such as myocardial infarction or stomach bleeding.

Regarding vascular ultrasound measurements, we found that lower T50 values associated with increased cIMT and cfPWV in a small subgroup of PXE patients for whom these data were available. Increased cIMT and cfPWV are frequently observed in individuals affected by PXE and is caused by a combination of premature and accelerated atherosclerosis and extensive medial vascular calcification (i.e., arteriosclerosis) [23,44,45]. Earlier studies identified T50 as a significant predictor of arterial stiffness in individuals with arterial hypertension or CKD, while baseline T50 was also shown to be independently associated with the progression of arterial stiffening over a 30-month follow-up period in predialysis CKD patients [34,46]. While our current findings need to be validated first in larger PXE patient cohorts, we can hypothesize that T50 may potentially be a reliable predictor of arterial stiffness (as determined using cfPWV) in PXE, which in itself is significantly associated with cardiovascular morbidity and mortality in humans [47].

No significant association between T50 and the total body CT calcium score was found, hence, suggesting that this radiological marker of vascular calcification burden does not fully grasp the extent of PXE disease severity as it only reflects the arteriosclerotic and not the noncalcific atherosclerotic component of PXE vasculopathy in contrast to the Phenodex scoring system, which provides information on clinically relevant endpoints such as the presence of debilitating symptoms like intermittent claudication or the need for vascular surgical interventions. As such, the associations between T50 and Phenodex scores identified in this study further indicate the capability of T50 to act as an important biomarker in PXE, reflecting actual and clinical disease severity, and might prove to be relevant in the follow-up, risk stratification and management of PXE patients as well as in future therapeutic trials.

Regarding a possible pathophysiological link between T50 and PXE disease severity, in vitro studies have shown that exposing vascular smooth muscle cells to secondary CPPs provokes a substantial inflammatory and procalcifying reaction, and increases oxidative stress [48,49]. As oxidative stress and increased cytokine production—as part of an overlapping process of excessive DNA damage response and premature senescence—have recently been shown to be involved in PXE pathogenesis, the dose-dependent damaging effects of secondary CPPs (as reflected by T50) might directly link T50 to the molecular process of PXE-related ectopic calcification [32,50].

In conclusion, this is the first study investigating serum calcification propensity T50 in a cohort of patients with a Mendelian ectopic calcification disorder, that is, PXE. The strengths of this study include the well-phenotyped patient group, hence, allowing adequate correction for potential confounders, and the relatively large sample size considering the rarity of the disease (population prevalence of PXE: 1/25,000–1/100,000 [22]). As serum T50 is considered a biological continuum without clearly identifiable or specific cut-off values, no direct comparison with healthy control individuals was performed in this study, similar to all previously performed research on serum T50 in other patient populations [12,34,51]. Limitations include the cross-sectional evaluation of T50 at a single time point and the fact that PXE patients were recruited from only one center. These issues should, therefore, be further addressed in larger, multinational and prospective studies evaluating changes in T50 over time and the potential link with disease progression in PXE patients.

## Figures and Tables

**Table 1:** Baseline characteristics of the study cohort.

	All	T50 Tertile 1 (<316 min)	T50 Tertile 2 (316–368 min)	T50 Tertile 3 (>368 min)	Tertile Comp. *p* Value	Correlation Coefficient	Correlation *p* Value
Number of participants, n	57	19	19	19			
Age (years)	45.2 ± 15.7	50.7 ± 12.0	43 ± 15.5	41.9 ± 18.3	0.17	−0.28	0.033 *
Sex, % female (n)	68.4 (39)	52.6 (10)	94.7 (18)	57.9 (11)	0.010 *	−0.01	0.94
Current smoking, % (n)	14.0 (8)	15.8 (3)	21.1 (4)	5.3 (1)	0.36	−0.16	0.25
Arterial hypertension, % (n)	17.5 (10)	21.1 (4)	15.8 (3)	15.8 (3)	0.89	−0.03	0.85
Hypercholesterolemia, % (n)	56.1 (32)	52.6 (10)	68.4 (13)	47.4 (9)	0.40	−0.06	0.64
Diabetes mellitus, % (n)	1.8 (1)	5.3 (1)	0 (0)	0 (0)	0.36	−0.17	0.20
Body mass index (BMI) (kg/m^2^)	25.4 ± 5.0	25.3 ± 5.8	26.1 ± 4.7	24.9 ± 4.6	0.78	−0.15	0.26
Serum calcium (mmol/L)	2.38 ± 0.11	2.38 ± 0.10	2.38 ± 0.11	2.37 ± 0.13	0.90	0.004	0.98
Serum magnesium (mmol/L)	0.84 ± 0.06	0.84 ± 0.06	0.82 ± 0.07	0.87 ± 0.04	0.051	0.20	0.14
Serum phosphorus (mmol/L)	1.12 ± 0.17	1.18 ± 0.20	1.12 ± 0.14	1.05 ± 0.13	0.043 *	−0.34	0.009 *
Serum creatinine (mg/dL)	0.82 ± 0.15	0.81 ± 0.18	0.78 ± 0.13	0.87 ± 0.15	0.26	0.13	0.35
eGFR (ml/min/1.73 m^2^)	95.4 ± 20.2	94.5 ± 18.0	95.8 ± 23.5	96.0 ± 20.0	0.97	0.11	0.41
Serum alkaline phosphatase (U/L)	70.5 ± 19.2	69.0 ± 16.4	71.4 ± 19.7	71.1 ± 21.9	0.92	0.09	0.50
Serum fetuin-A (µg/mL)	17.0 ± 3.1	15.3 ± 1.7	16.8 ± 3.2	18.8 ± 3.2	0.001 *	0.44	<0.001 *
Plasma inorganic pyrophosphate (PPi) (µmol/L)	0.50 ± 0.16	0.53 ± 0.17	0.51 ± 0.17	0.46 ± 0.15	0.35	−0.15	0.26
Phenodex score							
Skin (S)	2 (1.5–2)	2 (2–2)	2 (2–2)	2 (1–2)	0.69	−0.11	0.40
Eye (E)	2 (2–3)	3 (2–3)	2 (2–2.5)	2 (2–2)	0.041 *	−0.47	<0.001 *
Vascular (V)	1 (0–1)	1 (1–1)	0 (0–1)	0 (0–1)	0.093	−0.38	0.003 *
Cardiac (C)	0 (0–0)	0 (0–0)	0 (0–0)	0 (0–0)	0.35	−0.12	0.36
Gastrointestinal (G)	0 (0–0)	0 (0–0)	0 (0–0)	0 (0–0)	NA	NA	NA
Renal (R)	0 (0–1)	0 (0–1)	0 (0–0.5)	0 (0–0.75)	0.67	−0.09	0.51
Cumulative (Cu)	5 (4–7)	6.5 (6–7)	5 (4–5.5)	4 (3.25–5)	0.014 *	−0.47	<0.001 *
*ABCC6* genotype					0.93	−0.03	0.86
LOF + missense, % (n)	42.1 (24)	36.8 (7)	52.6 (10)	36.8 (7)	NA	NA	NA
Missense + missense, % (n)	15.8 (9)	15.8 (3)	15.8 (3)	15.8 (3)	NA	NA	NA
LOF + LOF, % (n)	26.3 (15)	31.6 (6)	15.8 (3)	31.6 (6)	NA	NA	NA
Other (single or splice), % (n)	15.8 (9)	15.8 (3)	15.8 (3)	15.8 (3)	NA	NA	NA
cIMT (mm) (n = 16)	0.64 ± 0.12	0.75 ± 0.13	0.63 ± 0.11	0.56 ± 0.08	0.068	−0.57	0.022 *
cfPWV (m/s) (n = 14)	8.7 ± 2.4	11.2 ± 1.8	9.0 ± 2.5	7.0 ± 1.08	0.11	−0.61	0.020 *
CT calcium score (Agatston units) (n = 14)	542 (4–2189)	1091 (496–4537)	524 (260–5001)	2 (0–724)	0.45	−0.45	0.10

Demographic, clinical, imaging and biochemical data from all PXE patients (n = 57) included in the study are shown, stratified by T50 tertile. cfPWV—carotid–femoral pulse wave velocity; cIMT—carotid intima–media thickness; comp.—comparison; CT—computed tomography; eGFR—estimated glomerular filtration rate; LOF—loss-of-function variants. * *p* < 0.05.

**Table 2:** Multivariate model of determinants of serum calcification propensity T50 in PXE. Age, serum phosphorus and serum magnesium levels were independent determinants of T50 in PXE patients. ANOVA—analysis of variance; SE—standard error. * *p* < 0.05.

	Unstandardized Coefficients		Standardized Coefficients	T Value	*p* Value
	Beta	SE	Beta		
T50 (final model)					
(Constant)	107.4	125.0			
Serum fetuin-A	8.67	2.49	0.40	3.49	<0.001 *
Serum phosphorus	−132.1	46.8	−0.32	−2.82	0.007 *
Serum magnesium	283.9	130.7	0.25	2.17	0.034 *
Model summary	R	Adjusted R^2^	ANOVA: F value	ANOVA: *p* value	
	0.58	0.30	8.80	<0.001 *	

**Table 3:** **Multivariate models of determinants of Phenodex Eye, Vascular and Cumulative scores in PXE.** Serum T50 independently and inversely associated with ophthalmological, vascular and overall disease severity in PXE patients. SE—standard error. * *p* < 0.05.

**Phenodex Eye** **(Final Model)**	**Parameter Estimates**		***p* Value**	**Model Fitting**	
	Estimate	SE		Chi-square	*p* Value
				33.4	<0.001 *
Age	0.084	0.026	0.001 *		
T50	−0.014	0.006	0.013 *		
Serum fetuin-A	−0.25	0.13	0.044 *		
**Phenodex Vascular** **(Final Model)**	**Parameter Estimates**		***p* Value**	**Model Fitting**	
	Estimate	SE		Chi-square	*p* value
				11.3	0.004 *
Arterial hypertension	1.42	0.69	0.039 *		
T50	−0.010	0.004	0.013 *		
**Phenodex Cumulative** **(Final Model)**	**Parameter Estimates**		***p* Value**	**Model Fitting**	
	Estimate	SE		Chi-square	*p* value
				16.2	0.001 *
Age	0.025	0.017	0.15		
Serum fetuin-A	−0.11	0.088	0.23		
T50	−0.010	0.004	0.016 *		

## Data Availability

Original data used in this study are available upon reasonable request to the corresponding author.

## Supplementary Materials

The following supporting information can be downloaded at: https://www.mdpi.com/article/10.3390/jcm11133727/s1, Figure S1: associations of serum calcification propensity T50 with vascular ultrasound measurements and total body CT calcium score in PXE; Table S1: overview of all PXE patients (n = 57) studied; Table S2: multivariate models of determinants of carotid intima–media thickness and carotid–femoral pulse wave velocity in PXE.
