# Supplementary material for: Serum Calcification Propensity T50 Associates with Disease Severity in Patients with Pseudoxanthoma Elasticum
Source: J Clin Med. 2022 Jun 28;11(13):3727. doi: 10.3390/jcm11133727 (PMC9267205; doi:10.3390/jcm11133727)
Supplement: Supplementary file 1 [file jcm-11-03727-s001.zip › jcm-1742689-supplementary.pdf]

Supplemental Table S1. Overview of all PXE patients (n=57) studied. *ABCC6* RefSeq NM_001171.5; genome build GRCh38 (hg38). C=Phenodex Cardiac score, cDNA=complementary DNA, Cu=Phenodex Cumulative score, E=Phenodex Eye score, F=female, G=Phenodex Gastro-intestinal score, gDNA=genomic DNA, ID=identification, M=male, R=Phenodex Renal score, S=Phenodex Skin score, V=Phenodex Vascular score.

Patient ID	Age *(years)*	Sex	Serum T50 *(minutes)*	*ABCC6* allele 1 *(cDNA/gDNA)*	*ABCC6* allele 1 *(protein)*	*ABCC6* allele 2 *(cDNA/gDNA)*	*ABCC6* allele 2 *(protein)*	S	E	V	C	G	R	Cu
01	25	F	408	c.3364delT	p.S1122Lfs*33	c.3364delT	p.S1122Lfs*33	1	2	1	0	0	0	4
02	80	F	285	c.2279G>A	p.R760Q	c.3421C>T	p.R1141*	1	4	1	0	0	/	/
03	73	F	323	c.3188T>G	p.L1063R	c.3188T>G	p.L1063R	2	3	0	0	0	0	5
04	43	F	324	c.3364delT	p.S1122Lfs*33	c.1194C>G	p.S398R	3	2	0	0	0	1	6
05	30	F	275	c.3669G>A	p.W1223*	c.3669G>A	p.W1223*	3	3	1	0	0	0	7
06	31	M	462	c.3421C>T	p.R1141*	c.3421C>T	p.R1141*	2	2	0	0	0	0	4
07	20	F	359	c.2278C>T	p.R760W	g.65280_73844del		0	2	0	0	0	0	2
08	25	F	364	c.2278C>T	p.R760W	g.65280_73844del		2	2	0	0	0	0	4
09	16	F	415	c.3421C>T	p.R1141*	g.5001_78907del		2	1	0	0	0	0	3
10	33	M	420	c.3507-3C>A		c.2831C>T	p.T944I	3	2	0	0	0	1	6
11	55	M	271	c.1132C>T	p.Q378*	c.1132C>T	p.Q378*	2	3	2	0	0	1	8
12	46	F	327	c.1553G>A	p.R518Q	c.1553G>A	p.R518Q	2	2	0	0	0	1	5
13	24	M	358	c.3506+2T>C		g.5001_78907del		2	2	0	0	0	1	5
14	57	F	318	c.1552C>T	p.R518*	c.3421C>T	p.R1141*	2	3	3	0	0	0	8
15	80	F	387	c.1552C>T	p.R518*	c.3421C>T	p.R1141*	1	3	0	0	0	0	4
16	64	M	407	c.3421C>T	p.R1141*	c.1108A>G	p.N370D	0	2	0	0	0	0	2
17	68	F	442	c.3422G>A	p.R1141Q			3	0	0	0	0	0	3
18	28	F	326	c.3421C>T	p.R1141*	c.3907G>C	p.A1303P	2	2	0	0	0	0	4
19	67	M	401	c.3188T>G	p.L1063R	c.3437T>C	p.F1146S	1	2	1	0	0	0	4
20	59	F	481	c.1132C>T	p.Q378*	c.1171A>G	p.R391G	2	2	1	0	0	0	5
21	49	F	332	c.3421C>T	p.R1141*	c.3437T>C	p.F1146S	1	2	1	0	0	0	4
22	35	F	499	c.3421C>T	p.R1141*	c.3437T>C	p.F1146S	2	2	0	0	0	0	4

23	46	F	389	c.3421C>T	p.R1141*	c.3188T>G	p.L1063R	1	2	1	0	0	0	4
24	54	M	260	c.3902C>T	p.T1301I			0	2	1	0	0	0	3
25	48	M	301	c.3364delT	p.S1122Lfs*33	c.1552C>T	p.R518*	2	2	2	0	0	1	7
26	39	F	378	c.2304C>A	p.Y768*	c.3421C>T	p.R1141*	1	2	0	0	0	0	3
27	51	M	411	c.3907G>C	p.A1303P	c.1636-11_1636-10delinsAG		2	3	2	2	0	1	10
28	30	F	341	c.3421C>T	p.R1141*	c.2432C>T	p.T811M	2	2	0	0	0	0	4
29	31	F	432	c.3940C>T	p.R1314W	c.3940C>T	p.R1314W	2	2	0	0	0	1	5
30	41	M	448	c.3907G>C	p.A1303P	g.65280_73844del		2	3	1	0	0	1	7
31	29	M	409	c.3421C>T	p.R1141*	c.3421C>T	p.R1141*	0	2	0	0	0	0	2
32	66	F	273	c.3775delT	p.Y1259Mfs*33	c.3775delT	p.Y1259Mfs*33	2	4	1	0	0	0	7
33	47	F	323	c.3421C>T	p.R1141*	c.1996G>T	p.G666V	2	2	1	0	0	0	5
34	39	F	374	c.3421C>T	p.R1141*	c.1996G>T	p.G666V	3	2	1	0	0	1	7
35	59	F	173	c.3412C>T	p.R1138W	c.3421C>T	p.R1141*	2	4	1	2	0	1	10
36	58	M	315	g.65280_73844del		g.65280_73844del		2	3	1	0	0	1	7
37	48	F	315	g.1664_72917del		c.4153G>C	p.A1358P	3	2	3	0	0	0	8
38	53	F	239	c.3421C>T	p.R1141*	g.65280_73844del		2	2	1	0	0	0	5
39	20	M	397	c.1553G>A	p.R518Q	c.1553G>A	p.R518Q	2	2	0	0	0	/	/
40	53	F	275	c.3907G>C	p.A1303P	c.3421C>T	p.R1141*	3	3	0	0	0	0	6
41	52	F	277	c.1944-1G>C		c.3907G>C	p.A1303P	2	3	0	0	0	0	5
42	49	M	276	c.1944-1G>C		c.3907G>C	p.A1303P	2	3	1	0	0	0	6
43	44	F	308	c.1552C>T	p.R518*	c.3662G>A	p.R1221H	2	3	1	0	0	1	7
44	31	F	364	g.65280_73844del		c.1892_1943+26del78	p.I631fs*1	3	2	0	0	0	0	5
45	44	M	248	c.4213G>A	p.G1405S	c.3074T>C	p.L1025P	2	3	0	0	0	1	6
46	61	F	327	c.3421C>T	p.R1141*	c.1355C>A	p.A452D	3	2	3	0	0	1	9
47	37	M	313	c.3941G>A	p.R1314Q	g.65280_73844del		2	2	1	0	0	1	6
48	36	F	322	c.3941G>A	p.R1314Q	g.65280_73844del		2	2	1	0	0	0	5
49	55	F	368	c.3389C>T	p.T1130M	c.3907G>C	p.A1303P	2	2	1	0	0	0	5
50	61	M	281	c.2252T>A	p.M751K	c.3907G>C	p.A1303P	0	3	3	2	0	0	8
51	22	F	486	c.3421C>T	p.R1141*	c.1171A>G	p.R391G	2	2	0	0	0	0	4
52	51	F	359	c.2782G>A	p.G928S			0	3	0	0	0	0	3

53	63	F	361	c.3421C>T	p.R1141*	c.3907G>C	p.A1303P	2	3	1	0	0	0	6
54	30	F	297	c.3421C>T	p.R1141*	c.3907G>C	p.A1303P	2	2	1	0	0	0	5
55	53	F	349	c.3421C>T	p.R1141*	c.3421C>T	p.R1141*	3	3	2	0	0	1	9
56	43	M	248	c.3941G>A	p.R1314Q	c.1171A>G	p.R391G	3	2	1	0	0	0	6
57	25	F	351	c.2420G>A	p.R807Q	c.2787+1G>T		1	2	1	0	0	0	4

Supplemental Table S2. Multivariate models of determinants of carotid intima-media thickness and carotid-femoral pulse wave velocity in PXE. Serum T50 independently and inversely associates with cIMT (n=16) and cfPWV (n=14) in PXE patients. eGFR=estimated glomerular filtration rate, SE=standard error. *$p<0.05$.

	**Unstandardized coefficients**		**Standardized coefficients**	**T value**	**P value**
	Beta	SE	Beta		
**Carotid intima-media thickness (final model)**					
(Constant)	0.914	0.113			
T50	-0.001	0.00	-0.50	-2.76	*0.016**
Hypertension	0.153	0.055	0.51	2.81	*0.015**
**Carotid-femoral PWV (final model)**					
(Constant)	19.406	1.918			
T50	-0.011	0.005	-0.32	-2.33	*0.042**
eGFR	-0.078	0.017	-0.67	-4.63	*<0.001**

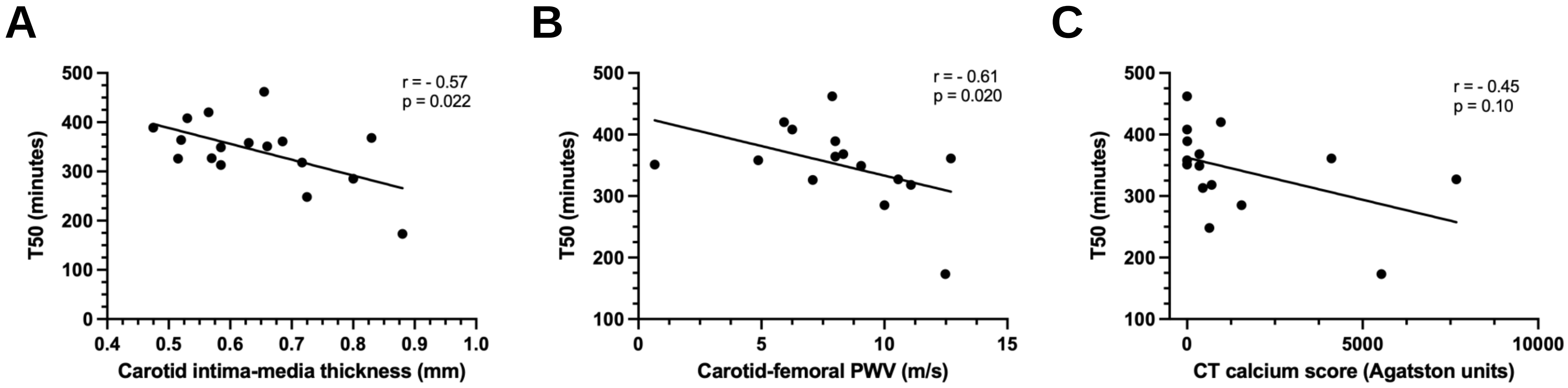

Supplemental Figure S1. Associations of serum calcification propensity T50 with vascular ultrasound measurements and total body CT calcium score in PXE. Serum T50 inversely correlates with carotid intima-media thickness (n=16; panel A) and carotid-femoral pulse wave velocity (n=14; panel B) in PXE patients, but not with total body CT calcium score (n=14; panel C). CT=computed tomography, PWV=pulse wave velocity.
